# Three Cases of Resection of Alpha-Fetoprotein-Positive High-Grade Fetal Adenocarcinoma of the Lung From a Single Institution

**DOI:** 10.7759/cureus.103792

**Published:** 2026-02-17

**Authors:** Yusuke Nabe, Hiroshi Mizuuchi, Masaaki Inoue, Junichi Yoshida

**Affiliations:** 1 Department of Chest Surgery, Shimonoseki City Hospital, Yamaguchi, JPN

**Keywords:** alpha-fetoprotein, biomarkers, cancer therapy, immunohistochemical analyses, lung cancer

## Abstract

We describe three cases of resection of embryonal lung adenocarcinoma, along with a literature review and immunohistochemical analyses of alpha-fetoprotein (AFP) levels. Generally, embryonal lung adenocarcinoma has a poor prognosis, which can be attributed to low programmed death-ligand 1 (PD-L1) expression, limiting the efficacy of immune checkpoint inhibitors. The cases showed varying intensities of AFP staining in the tumor tissues. Case 1 was initially negative for AFP, but positive at low levels upon re-staining; Case 2 was positive in a relatively small subset of cells; and Case 3 was intensely positive. Further, Case 3 showed elevated serum levels of AFP. Only Case 2 was positive for PD-L1 at relatively low levels. Patient 1 achieved >50-month overall survival, which can be attributed to early detection and complete resection. Cases 2 and 3 showed a poor prognosis. Our findings suggested that low or absent PD-L1 and moderate-to-high AFP expression may contribute to poor prognosis. AFP may train dendritic cells to promote differentiation of naïve T-cells to regulatory T-cells in the tumor microenvironment, which protects the tumor from adaptive immune responses. Therefore, combination therapy involving an AFP-targeted drug and an immune checkpoint inhibitor may be a viable treatment strategy for AFP-positive fetal lung adenocarcinoma.

## Introduction

Fetal adenocarcinoma is a rare lung tumor that was originally named “pulmonary blastoma,” given its appearance as a biphasic lung tumor comprising an epithelial component surrounded by a mesenchymal stroma that resembles a fetal lung [1]. Subsequently, the term “well-differentiated fetal adenocarcinoma of the lung” was introduced for classifying other epithelial lung tumors presenting with the epithelial component observed in pulmonary blastoma but lacking the mesenchymal components [2]. Finally, the World Health Organization classified this tumor as a pulmonary subtype [1]. Fetal adenocarcinoma can be classified as high-grade and low-grade adenocarcinoma [3]. Fetal lung adenocarcinoma accounts for 0.1-0.5% of all lung cancers [1]; further, its prognosis and optimal treatment regimen remain unclear. It is relatively predominant among older male heavy smokers, with a mean two-year postoperative mortality of 40% [4]. The prognosis of lung adenocarcinoma containing high-grade fetal adenocarcinoma components has been reported to be as poor as that of micropapillary adenocarcinoma [5]. Moreover, the prognosis of pure high-grade fetal adenocarcinoma is very poor, with recurrence occurring within two years in most cases [4].

There are no reported cases of high-grade fetal lung adenocarcinoma with high expression of programmed death-ligand 1 (PD-L1), with low or absent PD-L1 expression being among the underlying causes of poor prognosis [5].

Alpha-fetoprotein (AFP) expression has been reported in some high-grade fetal lung adenocarcinomas [6]. AFP levels have demonstrated utility as a diagnostic biomarker and therapeutic target for hepatocellular carcinoma (HCC) [7]. Here, AFP may train dendritic cells to promote differentiation of naïve T-cells to regulatory T-cells in the tumor microenvironment, which protects the tumor from adaptive immune responses [8].

Nonetheless, in some AFP-producing lung carcinomas, serum AFP levels have been reported to rapidly normalize after tumor resection [9]. Furthermore, patients with AFP-negative hepatoid adenocarcinoma of the lung have been shown to survive several months longer than those with AFP-positive disease [10]. AFP may potentially be an effective biomarker for assessing treatment efficacy and prognosis in lung cancer. Accordingly, this article describes three cases of resection of fetal lung adenocarcinoma, as well as presents a literature review.

## Case presentation

**Figure 1:**
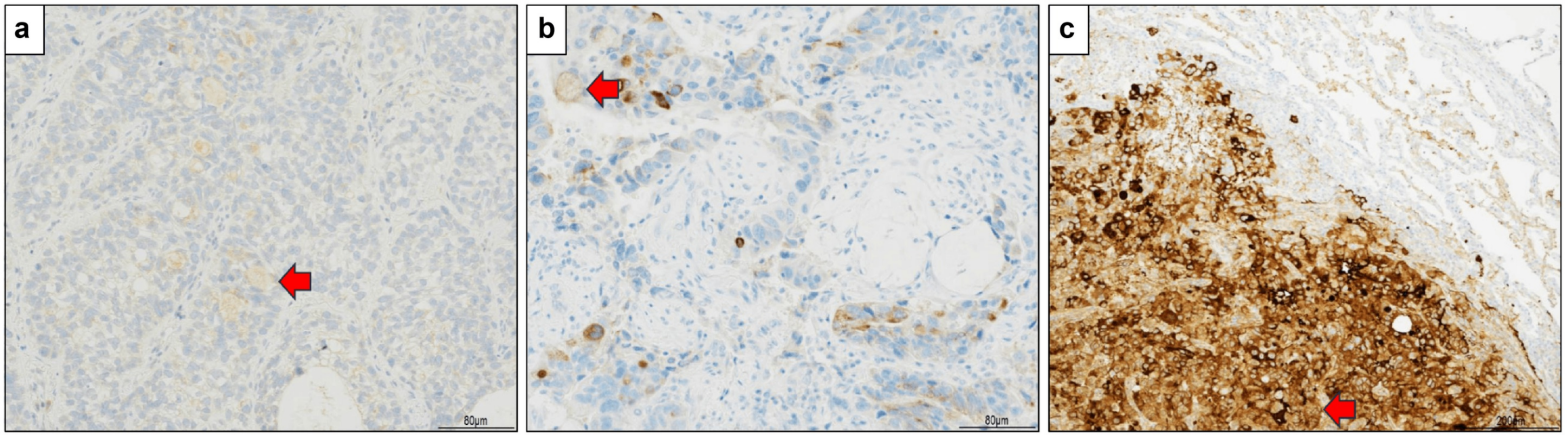
Alpha-fetoprotein (AFP) immunostaining for all cases. a. Case 1 (magnification: 100×). AFP was negative in the initial staining; however, when the positive antibody was changed and re-staining was performed, most of the tumor cells were weakly positive. b. Case 2 (magnification: 100×). A small number of cells were weakly positive. c. Case 3 (magnification: 40×). The result was strongly positive.

Diagnoses were confirmed by a single pathologist based on hematoxylin and eosin staining, as well as immunohistochemistry. Table 1 summarizes the three cases. Appendix 1 shows the immunohistochemistry results. AFP was positive in all cases. In Case 1, AFP (rabbit polyclonal; NICHIREI BIOSCIENCES Inc., Tokyo, Japan; product code: 422211) was not detected in the initial staining; accordingly, re-staining (rabbit polyclonal; Cell Marque, Rocklin, CA, USA; product code 518-101015) was performed. Figure 1 shows the AFP immunohistochemistry results. Immunohistochemistry was performed using a Ventana BenchMark XT automated staining system (Ventana Medical Systems, Inc., Roche, Tucson, AZ, USA). Heat-induced antigen retrieval was performed in an EDTA-based retrieval buffer at 95°C for 60 minutes.

**Table 1:** Patient characteristics. BI: Brinkman Index; M: months; PD-L1: programmed cell death 1-ligand 1; p-stage: pathological-stage; R0: none remaining; R2: macroscopic residual; TPS: tumor proportion score

Cases	Sex	Age	Chief complaint	BI	Diagnosis	Surgical procedure	TNM	p-stage	R	PD-L1; TPS	Recurrence	Survival	Survival period (M)
Case 1	Male	81 years	None	1960	Right upper lobe lung cancer	Right upper lobe partial resection	pT1bN0M0	IA2	R0	0%	None	Alive	50
Case 2	Male	74 years	Anterior chest pain	300	Left lower lobe lung cancer	Left lower lobectomy	pT3N2M0	IIIB	R0	25～49%	Lymph nodes	Death	15
Case 3	Female	75 years	None	364	Right upper lobe lung cancer	Right upper lobe partial resection	pT1cN2M1b	IVA	R2	0%	Not applicable	Death	16

Case 1

**Figure 2:**
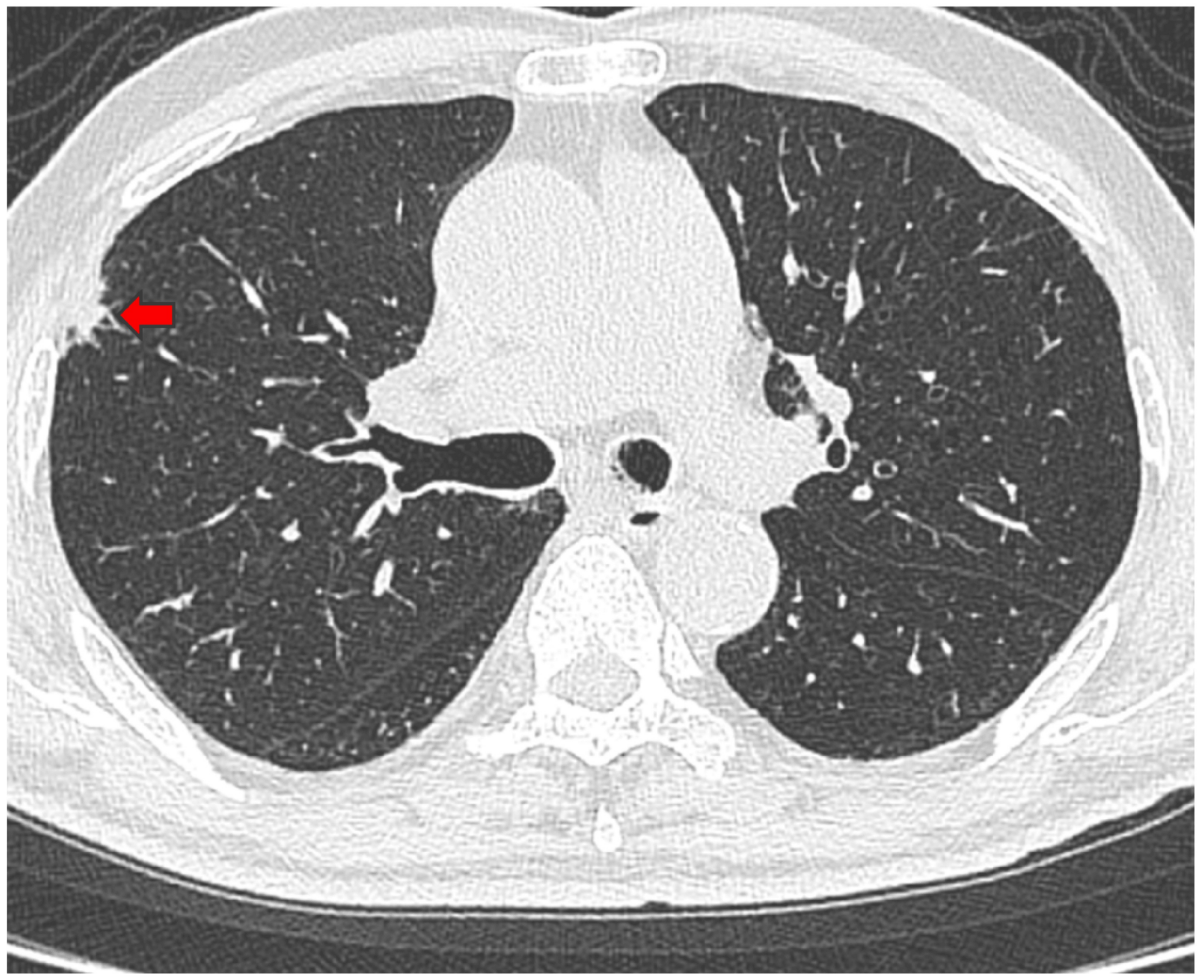
CT findings at the initial and last visits for Case 1. A 12 × 9 mm nodule was found in the right upper lobe. The arrow denoted the nodule.

An 81-year-old man was followed regularly by his family doctor for diabetes. Chest CT scan revealed a ground-glass opacity in the right upper lobe with a tendency to grow. He was referred to our department with a diagnosis of right upper lobe lung cancer. He had smoked 40 cigarettes per day between the ages of 20 and 69 years. Tumor marker levels were as follows: carcinoembryonic antigen (CEA) 7.0 ng/mL (normal range: 0-5 ng/mL), squamous cell carcinoma antigen (SCC) 0.9 ng/mL (normal range: ≤1.5 ng/mL), cytokeratin 19 fragment (CYFRA) 1.7 ng/mL (normal range: 0-3.5 ng/mL), sialylated Lex-i antigen (SLX) 24.9 U/mL (normal range: 0-38 U/mL), and pro-gastrin-releasing peptide (PRO-GRP) 45.5 pg/mL (normal range: 0-81 pg/mL). CT images are shown in Figure 2. Further examination revealed no obvious distant metastasis. A thoracoscopic partial resection of the right upper lobe was performed for both diagnosis and treatment. The right chest tube was removed on postoperative day (POD) one, and the patient’s condition improved. He was discharged home on POD five. Pathological diagnosis was high-grade fetal adenocarcinoma, pT1bN0M0 p-stage IA2. Postoperative adjuvant chemotherapy was not administered, and the patient was followed up as an outpatient. Four years and eight months after surgery, the patient is still alive and free of recurrence. In Case 1, serum AFP levels were not measured preoperatively but were measured after tumor resection and found to be negative.

Case 2

**Figure 3:**
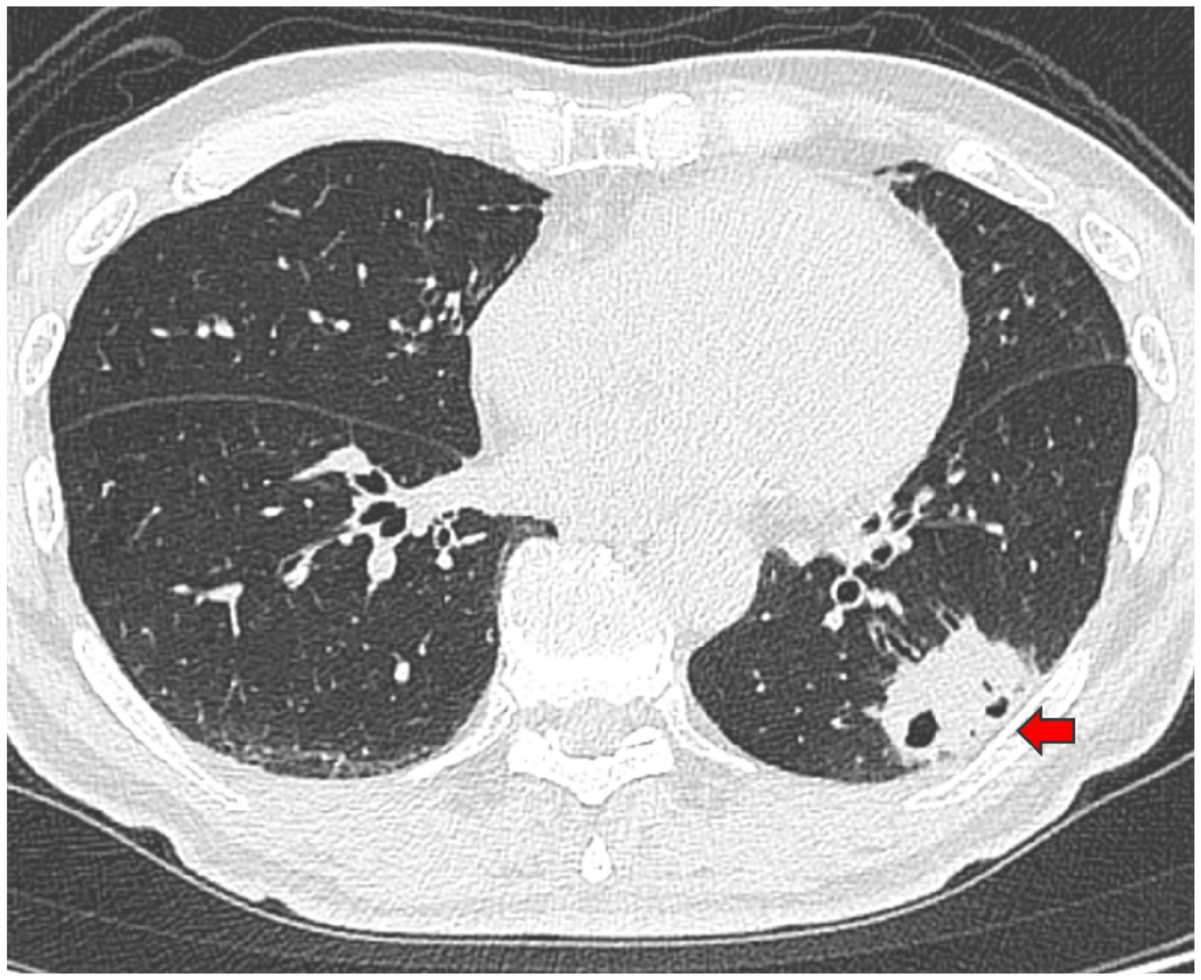
CT findings at the initial and last visits for Case 2. A 41 × 24 mm mass with a cavity was found in the left lower lobe. The arrow shows the mass with a cavity.

A 74-year-old man presented with right anterior chest pain and visited a local hospital. Chest X-ray revealed a nodular shadow in the left lower lung field. Chest CT revealed a mass shadow in the left lower lobe, and left lower lobe lung cancer was suspected. He was referred to our department. He had a history of smoking 10 cigarettes per day between the ages of 20 and 50 years. Tumor marker levels were as follows: CEA 2.9 ng/mL (normal range: 0-5 ng/mL), SCC 2.8 ng/mL (normal range: ≤1.5 ng/mL), CYFRA 2.3 ng/mL (normal range: 0-3.5 ng/mL), SLX 31.4 U/mL (normal range: 0-38 U/mL), and PRO-GRP 48.1 pg/mL (normal range: 0-81 pg/mL). CT images are shown in Figure 3. Further examination revealed no obvious distant metastasis. Preoperative bronchoscopy was performed, but no diagnosis was made. For both diagnosis and treatment, thoracoscopic left lower lobectomy and mediastinal lymph node dissection were performed. The left chest tube was removed on POD three, and the patient progressed well and was discharged home on POD seven. Pathological diagnosis revealed high-grade fetal adenocarcinoma, pT3N2M0 p-stage IIIB. Postoperative adjuvant chemotherapy with carboplatin plus docetaxel was administered. Lymph node recurrence was observed six months after surgery. In Case 2, serum AFP levels were not measured preoperatively but were measured after tumor resection and found to be negative.

Case 3

**Figure 4:**
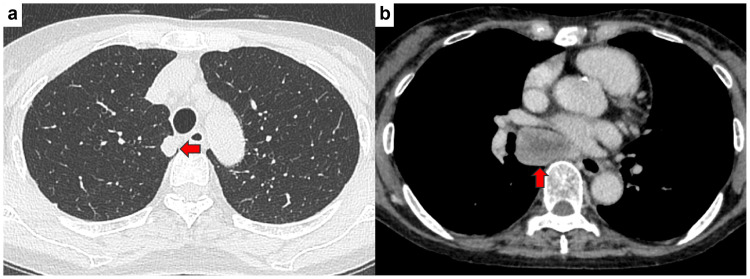
CT findings at the initial and last visits for Case 3. (a) A 16 × 12 mm nodule was found in the right upper lobe. The arrow denotes the nodule. (b) Lymph node enlargement was observed at the tracheal bifurcation, which suggested metastasis. The arrow denotes lymph node enlargement.

A 75-year-old woman was followed regularly in our hospital’s internal medicine department for rheumatoid arthritis. Chest CT scan revealed a nodule in the right upper lobe with a tendency to grow and enlarged subcarinal lymph nodes. She was referred to our department with suspected right upper lobe lung cancer. She had a history of smoking seven cigarettes per day between the ages of 22 and 74 years. Interstitial pneumonia was also noted as a comorbidity. Tumor marker levels were as follows: CEA 2.9 ng/mL (normal range: 0-5 ng/mL), AFP 2,096.1 ng/mL (normal range: 0.4-10 ng/mL), CYFRA 1.3 ng/mL (normal range: 0-3.5 ng/mL), SLX 27.3 U/mL (normal range: 0-38 U/mL), and PRO-GRP 24.4 pg/mL (normal range: 0-81 pg/mL). CT images are shown in Figure 4. Lymph node enlargement occurred below the tracheal carina, which suggested lymph node metastasis. Preoperative bronchoscopy was performed but was not diagnostic. For diagnostic purposes, a thoracoscopic partial right upper lobectomy and subcarinal lymph node biopsy were performed. The left chest tube was removed on POD four, and the patient progressed well and was discharged home on day 10. A head MRI taken after discharge revealed a metastatic brain tumor. Pathological diagnosis was high-grade fetal adenocarcinoma, pT1cN2M1b, p-stage IVA. Macroscopic incomplete resection was observed, and chemotherapy with atezolizumab, carboplatin, and nab-paclitaxel was initiated as primary treatment. In Case 3, the serum AFP level decreased to 764.9 ng/mL one month after surgery.

## Discussion

High-grade fetal adenocarcinoma is more common in men, with almost all patients being current or former smokers. Approximately half of the patients present with symptoms such as cough and bloody sputum [4]. In our case study, only one of the three patients exhibited symptoms. The two asymptomatic cases were incidentally discovered on CT scans while being followed for other illnesses. The symptomatic patient was diagnosed with pathological stage IIIB advanced lung cancer. Therefore, early detection during the asymptomatic period remains an issue.

Fetal lung adenocarcinoma typically presents with at least 50% fetal lung morphology, and high-grade fetal lung adenocarcinoma exhibits a lack of alveolar structures, as well as prominent nuclear atypia, prominent nucleoli, and frequent mitoses [11].

In addition to high-grade fetal adenocarcinoma, other types of lung cancer that produce AFP include AFP-producing lung carcinoma (mostly poorly differentiated adenocarcinoma) [9] and hepatoid adenocarcinoma of the lung, which exhibits hepatocyte-like differentiation [10]; however, their pathological appearance differs from that of fetal adenocarcinoma of the lung.

PD-L1 expression on tumor surfaces protects them from immune responses [12-14]. As a therapeutic strategy, immune checkpoint inhibitors (ICIs) reactivate the immune response by suppressing PD-L1 [15,16]. Notably, AFP enhances PD-L1 and B7-H4 expression, which may promote immune evasion by tumors [17].

In the present study, three and one cases were AFP-positive and PD-L1-positive, respectively; further, none of the cases showed >50% expression. Given the inter-organ differences in the immune environment [18], the relationship between AFP and PD-L1 may vary depending on the organ in which the cancer originates. Therefore, the relationship between AFP and PD-L1 may differ between AFP-positive fetal lung adenocarcinoma and HCC.

Although fetal lung adenocarcinoma generally has a poor prognosis [4], the long survival period observed in Case 1 can be attributed to the prompt complete resection. The other two patients underwent chemotherapy but died of the underlying disease. ICIs have limited therapeutic effectiveness against non-small-cell lung cancer (NSCLC) with PD-L1 expression <50% than against NSCLC with PD-L1 expression >50% (median overall survival (OS) for NSCLC with PD-L1 expression <1%, 1-49%, and ≥50% was 17.2, 21.8, and 27.7 months, respectively) [19].

In Case 2, ICI combined with chemotherapy was recommended; however, it was not administered due to work-related reasons and the patient’s refusal of hospitalization; accordingly, ICI monotherapy was instead administered on an outpatient basis. In patients with advanced NSCLC with a PD-L1 tumor proportion score (TPS) ≥20%, pembrolizumab alone achieved a better median OS than chemotherapy (18 vs. 13 months), with this benefit being smaller among patients with a TPS ≥50% (median OS: 20 months) [20]. In Case 2, the post-recurrence survival period was nine months, and the patient did not respond to ICI treatment. In Case 3, ICI combined with chemotherapy was administered, which achieved a survival period of 16 months.

HCC can be successfully treated with AFP-targeting drugs and ICIs [7]. AFP tends to suppress the immune system [7,8], which increases the tumor’s resistance to ICI treatment. Therefore, AFP is a potential therapeutic target.

There have been no reported cases of fetal lung adenocarcinoma with high PD-L1 expression [5]. In our case, PD-L1 expression was <50%, which suggested the potentially limited efficacy of ICI treatment. Further studies are warranted to evaluate the efficacy of combination therapy with an AFP-targeting drug and an ICI against AFP-positive fetal lung adenocarcinoma.

This study has some limitations. The mechanism of AFP-mediated immunosuppression was not directly demonstrated in this cohort and was primarily based on inference from the literature on HCC. Other limitations include a small sample size, a single-center design, and a lack of functional immune profiling analysis. Furthermore, the three cases differed in stage, tumor size, and treatment, which might have introduced bias.

## Conclusions

No cases of AFP-positive fetal lung adenocarcinoma exhibited high PD-L1 expression, and the therapeutic effect of ICIs was considered to be limited. Collectively, these findings suggest that drugs targeting AFP may potentially be an effective treatment strategy for AFP-positive fetal lung adenocarcinoma; however, further accumulation of cases and prospective trials is needed. Combination therapy involving an AFP-targeted drug and an ICI may be a viable treatment strategy for AFP-positive fetal lung adenocarcinoma.

## Appendices

 Appendix 1

**Table 2:** Immunostaining results. CD56: neural cell adhesion molecule 1; CEA: carcinoembryonic antigen; CgA: chromogranin A; GPC3: glypican3; hCG: human chorionic gonadotropin; p40: delta Np63; PAS: periodic acid-schiff; SYP: synaptophysin; TTF-1: thyroid transcription factor 1; U: unrated

Cases	PAS	GPC3	p53	CEA	TTF-1	hCG	CgA	SYP	CD56	p40
Case 1	+	+	−	U	+	U	+	+	+	+
Case 2	+	+	+	U	+	U	+	−	+	+
Case 3	U	+	+	+	−	−	−	−	−	U
